# Can the Clinical Utility of iBreastExam, a Novel Device, Aid in Optimizing Breast Cancer Diagnosis? A Systematic Review

**DOI:** 10.1200/GO.23.00149

**Published:** 2023-11-02

**Authors:** Fardeen Bhimani, Janice Zhang, Lamisha Shah, Maureen McEvoy, Anjuli Gupta, Jessica Pastoriza, Areej Shihabi, Sheldon Feldman

**Affiliations:** ^1^ Breast Surgery Division, Department of Surgery, Montefiore Medical Center, Montefiore Einstein Center for Cancer Care, New York, NY; ^2^ Albert Einstein College of Medicine, New York, NY

## Abstract

**PURPOSE:**

A portable, cost-effective, easy-to-use, hand-held Intelligent Breast Exam (iBE), which is a wireless, radiation-free device, may be a valuable screening tool in resource-limited settings. While multiple studies evaluating the use of iBE have been conducted worldwide, there are no cumulative studies evaluating the iBE's performance. Therefore this review aims to determine the clinical utility and applicability of iBE compared with clinical breast examinations, ultrasound, and mammography and discuss its strengths and weaknesses when performing breast-cancer screening.

**METHODS:**

A systematic review was performed following the Preferred Reporting Items for Systematic Reviews and Meta-Analyses guidelines. Four electronic databases were searched: PubMed, Cochrane Library, Web of Science, and Google Scholar.

**RESULTS:**

The review included 11 studies with a total sample size of 16,052 breasts. The mean age ranged from 42 to 58 years. The sensitivity and specificity of the iBE ranged from 34.3% to 86% and 59% to 94%, respectively. For malignant lesions, iBE demonstrated a moderate to higher diagnostic capacity ranging from 57% to 93% and could identify tumor sizes spanning from 0.5 cm to 9 cm.

**CONCLUSION:**

Our findings underscore the potential clinical utility and applicability of iBE as a prescreening and triaging tool, which may aid in reducing the burden of patients undergoing diagnostic imaging in lower- and middle-income countries. Furthermore, iBE has shown to diagnose cancers as small as 0.5 cm, which can be a boon in early detection and reduce mortality rates. However, the encouraging results of this systematic review should be interpreted with caution because of the device’s low sensitivity and high false-positive rates.

## Teaser

iBE, a radiation-free device using sensors, can aid in early breast cancer detection and reduce mortality in LMICs

## INTRODUCTION

Breast cancer is the most commonly diagnosed cancer worldwide, accounting for the severe burden, particularly among women.^1^ Early breast cancer detection significantly dictates mortality rates and treatment outcomes.^2-6^ A 3-month lag between symptom onset and treatment decreases the 5-year survival by 12%.^5^ In high-income countries (HIC), breast cancer is often detected early and has positive outcomes.^6^ In lower- and middle-income countries (LMICs), late presentation because of a lack of early clinical evaluation, screening, and diagnosis leads to underdetection, lower incidence, and higher mortality.^2,3,5^ Previous studies demonstrate that LMICs account for 53% of global breast cancer incidence and have 5-year survival rates of 40%-60% versus 80%-90% in HIC.^4,7^ Thus, early detection of breast cancer through screening is crucial to reducing mortality, especially in LMICs.

Although mammography and ultrasound are benchmarked breast cancer imaging tools, each has limitations. The high cost of mammography equipment and a scarcity of trained technologists and radiologists can exacerbate constraints on LMICs. Even in HIC, the sensitivity and specificity of mammograms are 87.8% and 90.5%, respectively.^8,9^ Women with dense breast tissue have even lower sensitivity numbers.^9-11^ By contrast, ultrasound is effective in detecting masses in dense breasts, but it is impractical for population-based screening because it is operator-dependent, has a high false-positive rate, and requires skilled ultrasound technologists and radiologist interpretation.

To bridge some of these concerns, a portable, cost-effective, easy-to-use, hand-held Intelligent Breast Exam (iBE) device has been developed as a point of care for breast examinations.^12-20^ This hand-held, wireless, radiation-free device uses sensors to electronically palpate breast tissue, which quantifies variations of tissue elasticity when pressed against the skin.^21,22^ The device detects abnormal tissue elasticity under the skin but cannot distinguish benign from malignant lesions.^13^

Multiple studies have been conducted highlighting iBE’s feasibility and diagnostic accuracy when compared with mammograms and ultrasounds.^12-20^ However, to the best of the authors’ knowledge, no cumulative study evaluating the iBE’s performance has been conducted. Therefore, we undertook a systematic review to evaluate the potential of the iBE device against benchmarked screening and imaging tools for detecting breast masses and assess its current strengths and limitations. As the iBE device continues to advance, it is important to summarize current technologies, benchmark its existing performance, and identify areas for improvement.

### Development of the Intelligent Breast Exam

**FIG 1:**
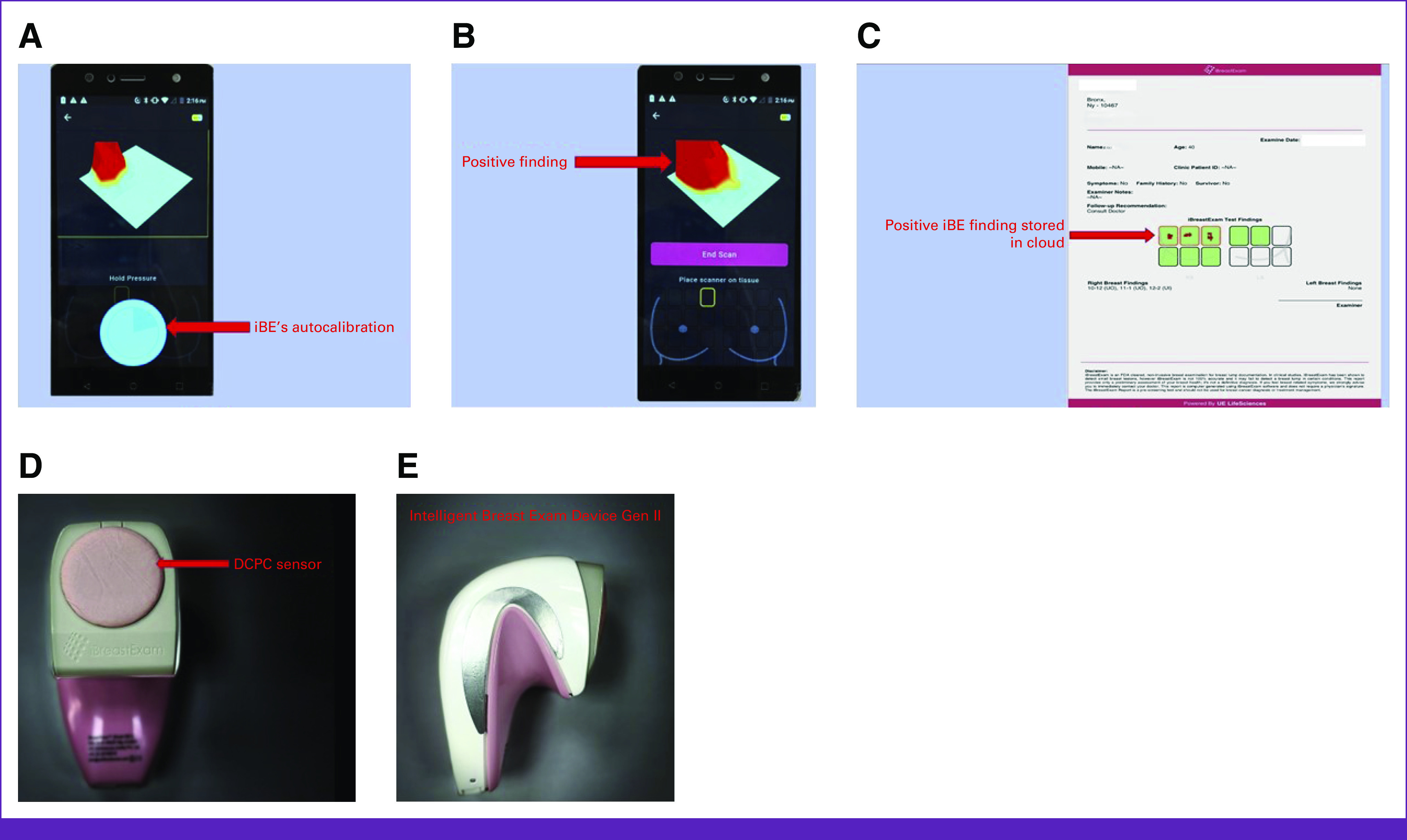
iBE device: (A) iBE's autocalibration technology. (B) iBE positive finding with the green area indicating normal and red corresponding to a suspicious lesion. (C) Positive iBE result stored in cloud. (D) iBE Gen II DCPC Sensor. (E) iBE Gen II device. DCPC, Dynamic Co-Planar Capacitive; iBE, Intelligent Breast Exam.

The US Food and Drug Administration approved the iBE device in 2013 to document clinical breast findings by measuring tissue compression using the piezoelectric finger (PEF), a tissue elasticity sensor that applies a direct current voltage.^13,16,23^ The device detects an induced voltage from tissue displacement caused by the applied voltage, mimicking palpation.^13,21^ A mass is directly detected by the contrast measured above the lesion compared with the surrounding tissue.^21^ PEF prototypes have been tested on breast phantoms and excised human tumors^21,24^ using a battery-powered iBE device with 4 × 4 fingers and a wireless mobile processor. On a 4 × 4 pressure array three-dimensional (3D) map, green represents normal tissue and red represents a suspicious lesion, emphasizing that no physician or operator interpretation is needed (Fig 1).^16,19^

The latest second-generation iBE system autocalibrates tissue signals using a hand-held compression probe with the 648 Dynamic Co-Planar Capacitive Sensor (DCPC Sensor) instead of piezoelectric sensors, a custom-built electronics board and a mobile phone (Fig 1).^23,25^ The iBreastExam Connect Mobile App wirelessly displays iBE device findings and stores and shares up to 10,000 breast examinations, making it easy to compare real-time results with past records (Fig 1).^16,25^

### Objectives

This review aims to determine the clinical utility and applicability of iBE compared with clinical breast examination (CBE), ultrasound, and mammography and discuss its strengths and weaknesses in optimizing breast cancer screening.

## METHODS

This systematic review was performed following the Preferred Reporting Items for Systematic Reviews and Meta-Analyses (PRISMA) guidelines.^26^ The study’s methodology was documented in a protocol registered in the PROSPERO database (number; CRD42022350723).

### Selection Criteria

A literature search was conducted from June to August 2022. Four electronic databases were searched: PubMed, Cochrane Library, Web of Science, and Google Scholar. The electronic search strategy included the following keywords: iBreastExam, iBreast[space]Exam, iBE, low resource, and piezoelectric fingers in breast cancer. In addition, we examined the bibliographic reference sections of the articles selected for this review to determine whether they met the inclusion criteria.

The authors discussed the inclusion criteria and reached a consensus. Research papers and electronically published research abstracts illustrating the use of iBE published in English with no date restrictions from the aforementioned databases were included. Furthermore, after identifying existing studies, all the collected articles were cross-checked to avoid duplicates.

**FIG 2:**
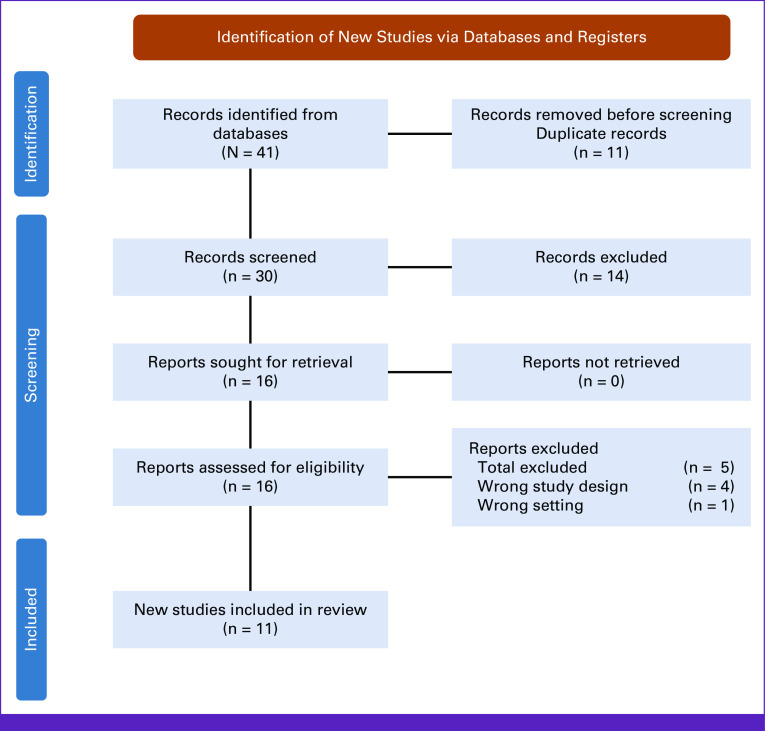
PRISMA flow diagram representing the selection process of the systematic review. PRISMA, Preferred Reporting Items for Systematic Reviews and Meta-Analyses.

Moreover, manuscripts were carefully examined and excluded using the following criteria: research articles (reviews, book chapters, conference reports, case reports, meta-analyses) and non-English articles were excluded. Two reviewers (F.B. and J.Z.) screened titles, abstracts, and full texts to find relevant articles that met eligibility criteria. PRISMA flowcharts were used to document the screening process (Fig 2).^26^

### Data Extraction

Two reviewers (J.Z. and L.S.) extracted the data into a structured Microsoft Excel spreadsheet. The following study characteristics were extracted from the included articles: study title, author name, year of publication, study design, and study region. Patient demographics, sensitivity and specificity percentages, iBE accuracy compared with CBE, and diagnostic imaging were all recorded.

### Quality Assessment

To assess the scientific quality of the studies included in our review and any possible source of bias, we prepared a checklist of questions in accordance with Quality Assessment of Diagnostic Accuracy Studies (QUADAS-2) guidelines.^27^ Four domains were used to identify applicability concerns and risk for bias: patient selection, the performance of index test, the standard of reference, and flow and timing. Any disagreements were resolved through a group discussion and consensus review. Each study was given a final designation of low or high bias on the basis of these categories.

### Data Synthesis

On the basis of the available data, a systematic review (qualitative synthesis) was conducted; however, because some of the included studies failed to report their diagnostic 2 × 2 table numbers and the remaining studies demonstrated high heterogeneity, a meta-analysis (quantitative synthesis) could not be performed.

## RESULTS

### Study Selection

The selection process of this systematic review is presented in the PRISMA flow diagram in Figure 2. Electronic search yielded 41 articles for screening. Of the 41 articles, 11 duplicate articles were removed. Thirty articles were screened, and 14 articles were found to be irrelevant (biotechnology review articles, studies not performing iBE examinations, commentary pieces). This screening process revealed 16 full-text articles and published abstracts, which were assessed for eligibility. Five studies were excluded, four for wrong study design and one for iBE use in the wrong setting. The final selection resulted in 11 studies included in this systematic review.

### Characteristics of Studies

This systematic review included 11 articles, which included an overall sample size of 8,025 women and one man, totaling 8,026 individuals and 16,052 breasts. The mean age of the individuals ranged from 42 to 58 years.^12-22^ The selected studies were geographically diverse, representing a total of five countries. Six of the 11 studies took place in LMICs (Brazil, Nigeria, and India), as determined by the 2022 World Bank classification.^28^ In terms of study design, all 11 studies were prospective and nonrandomized, assessing the efficacy of screening for breast cancer with the use of the iBreastExam and/or the PEF.^12-22^ All 11 studies were prospective and nonrandomized, testing the efficacy of iBE and/or PEF for breast cancer screening.^12-22^ Four studies were conducted in a screening setting and seven in a diagnostic and screening mix setting. Table 1 shows the demographic and clinical characteristics of the studies.

**TABLE 1:**
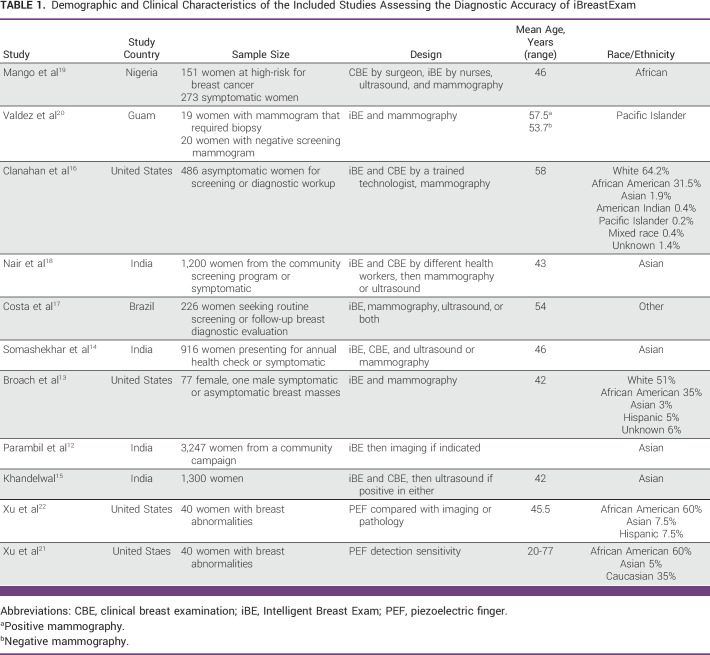
Demographic and Clinical Characteristics of the Included Studies Assessing the Diagnostic Accuracy of iBreastExam

Furthermore, 4 of the 11 studies compared iBE with CBE to determine a better screening technique for detecting breast lumps.^14,16,18,19^ Tables 2-4 show the results of comparison between iBE as a diagnostic technique versus Mammogram and/or ultrasound, CBE as a diagnostic technique versus Mammogram and/or ultrasound, and iBE versus CBE for identifying breast masses, respectively.

**TABLE 2:**
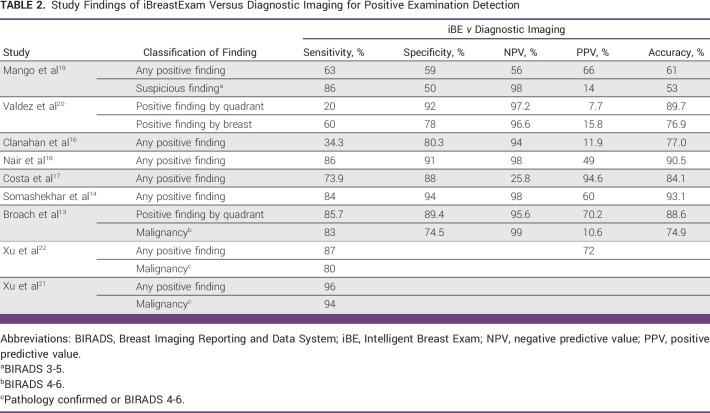
Study Findings of iBreastExam Versus Diagnostic Imaging for Positive Examination Detection

**TABLE 3:**
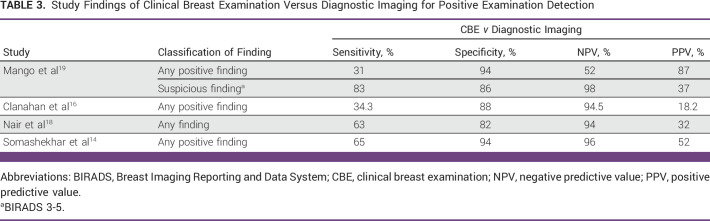
Study Findings of Clinical Breast Examination Versus Diagnostic Imaging for Positive Examination Detection

**TABLE 4:**
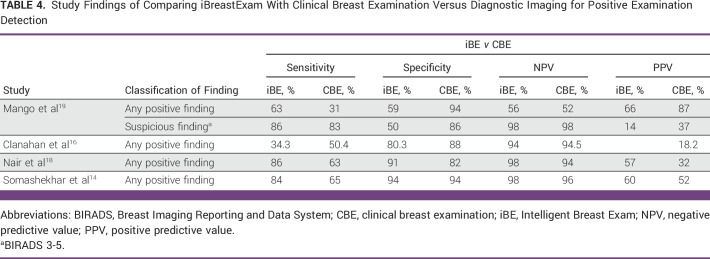
Study Findings of Comparing iBreastExam With Clinical Breast Examination Versus Diagnostic Imaging for Positive Examination Detection

In addition, on the basis of a 26-item breast health questionnaire, one study found a high patient acceptance of 67% for iBE on the basis of a 26-item breast health questionnaire.^20^ Similarly, studies performed in a hospital-based setting in Nigeria and a rural community-based study in India reported higher rates of patient satisfaction with the iBE device, accounting for 98% and 97%, respectively.^12,19^ Furthermore, despite the significantly lower cost of iBE scans compared with mammography, no study has performed a cost-effective analysis comparing it with gold standard imaging to assess its utility from the patient’s perspective.

### Summary of the Main Results

The clinical utility of PEF was first reported by Xu et al^22^ In their study of 40 patients with positive findings on CBE or imaging, PEF had an overall sensitivity of 87% compared with mammography, which increased to 100% in women younger than 40 years. PEF versus ultrasound yielded identical results. A follow-up study by the same group confirmed their findings in patients over 40 years, where PEF identified 46 of 48 lesions.^21^ Furthermore, 28 patients had multiple lesions, with PEF detecting 31 of 33 of all lesions (94%) and 14 of 15 of malignant lesions (93%), whereas mammography detected 30 of 33 of all lesions (91%) and 12 of 15 of malignant lesions (80%). They also demonstrated that breast density had no effect on PEF sensitivity.

Despite these promising results, studies have reported iBE sensitivity ranging from 34.3% to 86%.^13,14,16-20,22^ Clanahan et al^16^ reported the lowest sensitivity in a nonrandomized prospective study of 486 patients undergoing screening or diagnostic iBE, CBE, and mammography. Their study found 101 positive iBE results, 66 positive CBE results, and 35 positive mammograms results (Breast Imaging Reporting and Data System [BIRADS] 3,4, or 5) with a specificity of 80.3%, a false-positive rate (FPR) of 19.7%, and negative predictive value (NPV) of 94%. However, 12 patients in their cohort tested positive for iBE and mammography, yielding a sensitivity of merely 34.3%. Compared with iBE, CBE had better specificity (88%), FPR (12%), NPV (94.5%), and sensitivity (34.3%).

In Brazil, Costa et al^17^ recruited 226 women (449 breasts) for routine screening or follow-up breast diagnostic evaluation in a prospective nonrandomized trial. Clinicians were blinded, and iBE was compared with mammogram, ultrasound, and mammogram plus ultrasound to identify breast lesions. iBE had a sensitivity of 45.9% and a specificity of 87.7% compared with mammography and a sensitivity of 66.7% and a specificity of 87.8% compared with ultrasound. Compared with mammogram plus ultrasound, iBE’s sensitivity increased to 73.9%, but specificity remained unchanged (88%). Similarly, Somashekhar et al^14^ recruited 916 women from their annual health visits in India for iBE, CBE, and diagnostic imaging. A community health worker performed iBE, an expert clinician performed CBE, and a radiologist performed imaging in their triple-blinded study. Compared with imaging, they found that CBE had a sensitivity of 65%, a specificity of 94%, a positive predictive value (PPV) of 52%, and a NPV of 96%. CBE detected breast lesions in 314 women younger than 40 years with a sensitivity of 62%, a specificity of 92%, a PPV of 60%, and a NPV of 92%. Compared with breast imaging, iBE reported a sensitivity of 84%, a specificity of 94%, a PPV of 60%, and a NPV 98%. In 314 women younger than 40 years, iBE detected breast lesions with a sensitivity of 85%, a specificity of 93%, a PPV of 71%, and a NPV of 97%.

A unique approach for testing the iBE was opted by Valdez et al^20^ where they tested the device on patients and gelatin phantom cases. Thirty-nine women in their study had mammograms and were divided into two groups: positive (BIRADS diagnostic category 4 or higher) and negative. Overall, 19 lesions were found, with five being malignant, and iBE identified three of them, highlighting a sensitivity of 60% and a specificity of 78.1%. However, analysis by quadrants decreased the sensitivity to 20% and increased the specificity to 92.1%. In addition, for phantom cases, different gelatin material concentrations were used to depict tumor sizes from 2 to 25 mm and depths from 0.5 to 5 cm. Three hundred iBE tests on these phantom cases showed that the deepest tumor detection was 2.5 cm for 6% and 7% gelatin and the smallest tumor was 16 mm for 7%, 6%, 4%, and 3% gelatin.

Despite different study designs and settings, three studies, Mango et al,^19^ Nair et al,^18^ and Broach et al,^13^ showed similar iBE performance results. Mango et al^19^ recruited 424 Nigerian women with breast lumps, pain, discomfort, nipple discharge, axillary swelling, and skin changes or a high risk of breast cancer. They tested iBE to identify any breast finding and suspicious breast finding (defined as findings that require biopsy on the basis of breast imaging) and found that iBE had a sensitivity of 63%, a specificity of 59%, a NPV of 56%, and a PPV of 66%, whereas CBE had 31%, 94%, 52%, and 87%, respectively, compared with benchmarked imaging modalities. However, iBE’s diagnostic accuracy increased for suspicious breast finding with the sensitivity, specificity, NPV, and PPV of 86%, 50%, 96%, and 14%, compared with CBE’s 83%, 86%, 98%, and 37%. Nair et al^18^ found similar sensitivity among 1,263 women (2,526 breast) older than 30 years. In their study, they designated a positive finding as BIRADS 3-6, irrespective of mammography or ultrasound. Compared with diagnostic imaging, iBE had a sensitivity of 86%, a specificity of 91%, a NPV of 98%, and a PPV of 49%, whereas CBE had 63%, 82%, 94%, and 32%. Similarly, Broach et al^13^ reported similar sensitivity trends in their study of 77 women and one male participant. Because the PEF was designed to detect any finding, they defined a positive iBE finding as fibroadenoma, cyst, myofibroblastoma, fat necrosis, papilloma, ductal carcinoma in situ, or cancer. Similar to the study by Valdez et al,^20^ they examined breast quadrants; in reference to imaging, iBE demonstrated iBE had a sensitivity of 85.7%, a specificity of 89.4%, a NPV of 95.6%, and a PPV of 70.2%. However, Broach et al^13^ did not compare iBE with CBE or diagnostic imaging.

### Identification of Malignancy

Some studies assessed iBE’s accuracy in identifying cancerous lesions.^13,14,16,19-21^ Broach et al^13^ reported that 12 of the 78 patients in their study had cancer and 10 had positive iBE findings, demonstrating a sensitivity of 83% in detecting malignant lesions with a mean tumor size of 1.91 ± 0.9 cm. By contrast, Somashekhar et al^14^ found that iBE detected 8 of 8 malignant lesions and CBE detected 7 of 8 with an average lesion size of 1.5 cm. In addition, Clanahan et al^16^ demonstrated that both iBE and CBE could detect four of seven (57%) malignancies and one of the four iBE detected malignancy was missed by mammography. Moreover, iBE detected malignancies ranging from 1.2 to 3 cm but not tumors under 1 cm. Similarly, Valdez et al^20^ highlighted that iBE detected 60% of malignant tumors, with an average size of 1.6 ± 1.3 cm. Furthermore, Mango et al^19^ reported that iBE could identify 13 (87%) of the 15 malignant cases with an average tumor size of 3.3 cm, whereas Xu et al^21^ inferred that iBE could detect 93% (14 of 15) of the cancerous lesions with tumor sizes spanning from 0.5 to 9 cm. The results are further elaborated in Table 5.

**TABLE 5:**
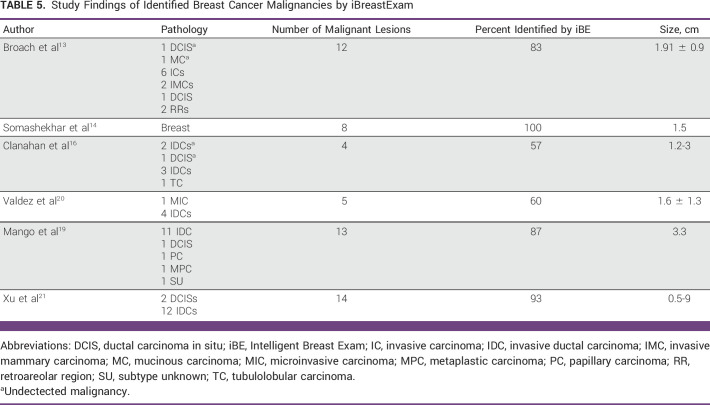
Study Findings of Identified Breast Cancer Malignancies by iBreastExam

## DISCUSSION

Over the past few decades, many developed countries have implemented population-based breast cancer screening programs, which have contributed to lower mortality and advanced cancer rates; however, the opposite is true for LMIC.^29-31^ Previous literature demonstrates that a variety of factors contribute to the existing health disparities in LMICs, ranging from a lack of health care facilities offering screening and diagnostic services to a limited number of well-trained care providers, and oftentimes psychosocial beliefs, cultural beliefs, and medical mistrust, which can also be obstacles to receiving timely cancer care.^32^ Astonishingly, many of these factors are also true for ethnically diverse patients who face socioeconomic disparities in HIC.^33,34^ To address these concerns, there are still ongoing research and debate on how to best implement breast cancer screening programs in resource-limited settings such as LMICs and patients experiencing socioeconomic disparities in HIC.

To alleviate some of these challenges, iBE may be a valuable screening tool in resource-constrained settings. The iBE is a radiation-free, cost-effective, and easy-to-use hand-held device that offers real-time results.^13,19,35^ Instead of requiring a competent expert or clinician to use and interpret the results, iBE can be used and interpreted after a quick training session.^19,23^ In patients younger than 40 years and with dense breast tissue, mammography sensitivity drops from 87% to 62.9%, a substantial downside.^10,11^ This is extremely crucial because breast density strongly correlates with an increased risk of breast cancer; a breast density core of >three fourths increases the risk of developing breast cancer by four to six folds.^36-41^ A majority of younger women, African American women, Asian women, and 43% of women age 40-74 years have been reported to have dense breasts.^42^ Contrastingly, in this regard, Xu et al^22^ inferred that their PEF technique detected 100% of breast lesions in patients 40 years or younger with highly dense breasts, making iBE a better alternative to mammography. However, drawing such strong conclusions is limited because of the small sample size. Moreover, there is a lack of consensus, despite multiple studies being conducted on the use of iBE because it has yielded mixed results in different settings. Therefore, this systematic literature review examines iBreastExam’s clinical utility and applicability to CBE and gold standard diagnostic imaging modalities including mammography and ultrasound.

Overall, iBE has demonstrated widespread patient acceptance. Three hospital and community-based studies—the study by Valdez et al^20^ in Guam, the study by Mango et al^19^ in Nigeria, and the study by Parambil et al^12^—in India reported moderate to higher rates of patient satisfaction with the iBE device (67%, 98%, and 97%, respectively). In addition, Mango et al^19^ reported that 98% of their patients had a painless iBE examination and 96% were willing to have an annual iBreastExam. Furthermore, another study conducted in rural India reported that it was easier to convince women to undergo the iBE screening compared with mammography, which is painful and radiation-intensive.^15^ Because the patients preferred iBE over mammography, it could become a popular rural prescreening device. However, the expense of such screening also influences patients’ desire to undertake diagnostic imaging. While manufacturers claim that iBE costs $5 per scan, which is nominal when compared with o mammography,^43^ there is currently no cost-effective study in the literature. A comprehensive cost-effectiveness analysis is recommended to assess the affordability and utility of iBE compared with mammography and ultrasound from the patients’ perspective.

Broadly, the reviewed studies have emphasized on the device’s ease of use and accessibility. With iBE’s autocalibration technology (Fig 1), numerous studies have used recent nursing school graduates, imaging technologists, and community health workers to administer examinations.^12-16,19^ Clanahan et al^16^ and Somashekhar et al^14^ further elaborate that although CBE may be cost-effective, participation rates may be low, particularly in resource-limited settings with few qualified CBE experts.^14,16^ Therefore, iBE is ideal for equipping community health workers with a standardized breast examination tool that eliminates human subjectivity and requires minimal training.^14,15^ In addition, electronic results from iBE simplify outreach organization and geographic tracking, which has directed follow-up imaging.^16^ In rural India, Parambil et al^12^ reported that 67.5% of patients reported for follow-up evaluation after receiving iBE results and counseling from community health workers.

A caveat associated with the use of mammography is an increased number of false alarms and overdiagnosis, which generally results in overtreatment and further increases the constraints on LMICs.^35^ However, using iBE as a triaging tool helps avoid this pitfall by reducing the number of patients referred for additional imaging because of its high negative predictive value (94%-99%) across six trials.^13,14,16,18-20^ This triaging method can be very helpful in LMICs where underfunded health care services are unable to screen all patients.^17^ Besides triaging, iBE data from five studies have demonstrated a high sensitivity of at least 83% and six studies have reported its specificity to be 88% or higher, suggesting that it could enhance breast cancer screening programs.^13,14,17-19,22^ This is further illustrated in Table 2. Moreover, nearly half of the studies in this review were conducted in symptomatic and high-risk patients, where iBE displayed higher sensitivity rates in most of these studies, suggesting that the device can be used in general screening programs and symptomatic patient screening.^13,19-22^

To further explore iBE's clinical utility in breast cancer diagnosis, many studies have described the device's potential in identifying malignant lesions.^13,14,16,19-21^ Most studies found diagnostic capability between 57% and 93%. In terms of tumor size, most of these studies reported that iBE could detect approximately 1- to 3-cm lesions, with the exception of Xu et al,^21^ demonstrating a wide range from 0.5 to 9 cm. However, all these studies had a relatively small sample size with malignant disease, Table 5. In addition, when detecting precancerous lesions, iBE's specificity plummeted to 50%, indicating higher numbers of false positives.^19^ These false-positive numbers become critical, especially in LMICs, since it consumes scarce diagnostic resources and generates an unnecessary cost burden with a minimal benefit to patients.^13^ At present, CBE is the only cost-effective prescreening modality in cancer care, but the USA's National Breast and Cervical Cancer Early Detection Program^44^ has published mediocre sensitivity (58.8%), which Barton et al^45^ corroborated by pooling data from six studies reporting a sensitivity of 54.1%. Similarly, in our review, four studies compared CBE against different imaging modalities, three of which showed iBE's advantage in diagnosing breast lesions (Table 4).^14,16,18,19^ Notably, all reviewed studies used the iBE Gen I device with PEF, with the exception of Mango et al^19^ who reported 192 examinations with the original device and 232 with an upgraded version, which improved calibration and specificity (reducing false positives) for suspicious findings (*P* < .0010).

Within the studies, the authors have noted some limitations in the clinical utility of iBE. Three studies, the studies by Clanahan et al,^16^ Valdez et al,^20^ and Broach et al,^13^ reported that the device failed to detect lesions <1 cm. Unlike other benchmarked imaging tests, iBE cannot show an image of the lesion, restricting its classification and making it unreliable for cancer diagnosis.^20^ In addition, a negative iBE test may sometimes create false hope and deter individuals from screening mammograms.^20^ Moreover, the use of iBE in dense breasts has been controversial, with Mango et al^19^ reporting higher sensitivity and specificity for nondense breasts compared with dense breasts, whereas Xu et al^21^ demonstrated that iBE's sensitivity was unaffected by breast density. As a result, further studies are warranted to determine iBreastExam's clinical utility in women with dense breasts.

The limitation of this review includes the overall smaller sample size of 11 studies, of which 8 have reported their sensitivity and specificity numbers, limiting the reliability of these results. In addition, there was significant heterogeneity across the studies, so pooled sensitivity and specificity estimates were not reported. Since the studies in this review were conducted in different parts of the world, variations in breast density among women from different geographical regions may have contributed to heterogeneity and inconsistent sensitivity percentages.^42^ Therefore, further studies are warranted for the use of iBE in different breast densities. Furthermore, in these studies, the number of positive patients was too small to predict the device's reliability in detecting breast lesions. In addition, there is a lack of data regarding the impact of iBE on mortality and the absence of a cost-benefit analysis, which may play a role in the decision-making process. Besides these limitations, subjective errors must be evaluated. Despite the device being used by social workers and doctors, none of these studies tested for inter-rater and intrarater reliability ratios to rule out subjective error. Finally, Mango et al^19^ reported that an upgraded device improved calibration and specificity in the latter phase of their study. Thus, future studies with a larger sample size having different breast mass sizes and densities are recommended using the newer iBE Gen II model to determine whether there are any improvements over its predecessor in diagnosing breast masses, which will further aid us in defining the potential efficacy of iBE.

In conclusion, our findings underscore the potential clinical utility and applicability of iBE as a prescreening and triaging tool, which may aid in reducing the burden of patients undergoing diagnostic imaging in LMICs. Furthermore, iBE has been shown to diagnose cancers as small as 0.5 cm, which can be a boon in early detection and reduce mortality rates. This finding, however, is based on a single small study, and the range of cancers diagnosed may vary. Furthermore, the effect on mortality has not been established. However, the encouraging results of this systematic review should be interpreted with caution because of the device's low sensitivity and high false-positive rates.
